# Smoking cessation among transit workers: beliefs and perceptions among an at-risk occupational group

**DOI:** 10.1186/s13011-015-0012-7

**Published:** 2015-05-13

**Authors:** Robynn S Battle, Carol B Cunradi, Roland S Moore, Valerie B Yerger

**Affiliations:** Prevention Research Center, Pacific Institute for Research and Evaluation, 180 Grand Avenue, Suite 1200, Oakland, CA 94612 USA; Department of Social and Behavioral Sciences, University of California, San Francisco, CA USA

**Keywords:** Transit workers, Smoking cessation, Beliefs, Perceptions, African American

## Abstract

**Background:**

Transit workers, in comparison to the general population, have higher rates of smoking. Although smoking cessation programs are often available through workers’ HMOs, these programs are frequently underutilized. Quitting practices, including participation in cessation programs, are often associated with beliefs about smoking behaviors and the ability to quit. We analyzed how transit workers’ beliefs about cessation might function as barriers to or facilitators of participating in cessation activities.

**Findings:**

We conducted 11 focus group discussions with 71 workers (45% female; 83% African American) at an urban public transit agency. Most participants (83%) were bus operators. Only current smokers and former smokers were recruited. Both current and former smokers recognized the need to quit and some were familiar with or at least aware of cessation programs and pharmaceutical aids offered through their HMO. Many, however, believed there were factors, such as smoker’s readiness to quit, recognition of the elements of addiction, and personal or observed experience with cessation, that facilitated or impeded successful quit attempts.

**Conclusion:**

Beliefs play an important role and influence the extent to which transit workers participate in smoking cessation. Being cognizant of and addressing these beliefs so that workers gain an informed understanding is important when designing cessation programs. Doing so may help in creating tobacco cessation efforts that are seen as both attractive and beneficial to transit workers.

## Findings

Workers in blue-collar occupations are significantly more likely to be current smokers than workers in white-collar occupations [1]. The age-adjusted prevalence of current smoking among workers in the transportation and material moving occupational group, which includes transit workers, is 28.7% [2]. Bus drivers and transit operators are exposed to an array of environmental work stressors, including traffic congestion, schedule-related time pressure, and hostile or violent passengers [3]. Work stressors are significantly associated with smoking behavior among transit workers [4]. For many, smoking is perceived as a coping strategy to mitigate work-related stress [5,6]. It should be noted that additional factors may contribute to the at-risk status of this occupational group. For example, although African Americans comprise 12% of the labor force, they constitute 25.3% of those employed as bus drivers in the U.S. [7]. This is salient because African Americans experience excessive rates of tobacco-related health consequences such as lung cancer compared to other racial/ethnic groups [8]. Although cessation programs are typically available to metropolitan transit workers through their health maintenance organization (HMO) as a union-negotiated employee benefit, elevated rates of smoking among this occupational group suggests these services may be underutilized [6]. Understanding health beliefs associated with cessation in this population may aid in encouraging the utilization of available cessation services or the creation of new ones that address transit workers’ specific needs.

This paper reports on focus groups that explored beliefs and perceptions about smoking cessation among transit workers, delving further into previously reported data investigating barriers to cessation among transit workers [6]. For example, the HMO coverage provided to the workers offers cessation programs for both smokeless and combustible tobacco use, and includes free telephone coaching sessions and quitline access, one-on-one individual coaching sessions, free group cessation classes, and cessation medication at the HMO’s drug benefit co-payment price when prescribed by their physician. Issues identified by transit workers as barriers to utilization of these programs included concerns about smoking cessation medication side effects, scheduling as a barrier to participation (i.e., conflicts due to work shift schedules), work-related fatigue, and concerns about confidentiality [6].

Theoretically, this study was influenced by three approaches to health behavior change: (1) the Health Belief Model, which focuses on predictors of health related decisions and behaviors that involve: a) perceived susceptibility towards the health issue in question; b) perceived severity of the condition; c) perceived benefits of the prescribed course of action; d) cues to action; and e) self-efficacy [9]; (2) the Social Cognitive Theory Model [10], which relies upon the influence of observed behaviors on one’s own beliefs about behavior change, and (3) the Transtheoretical Model, based upon the movement of people along stages or a continuum of readiness to change their behavior [11]. People’s personal beliefs about their health and current health practices constitute a strong indicator of the extent to which they will incorporate prevention and or intervention methods [12]. In the case of smoking cessation, beliefs about nicotine replacement therapy (NRT) or pharmaceuticals are indicators of whether a person might initiate medically assisted cessation efforts [13]. The findings from the current study can be used to inform the development of cessation programs that address their beliefs in order to maximize participation, thus reducing tobacco-related disparities among transit workers.

## Methods

Focus groups were conducted among a sample of urban transit workers to qualitatively explore their beliefs about smoking cessation. The urban transit workers for the study were recruited from AC Transit, a public transit agency based in Oakland, California. The target population consisted of bus operators and maintenance workers, all members of the same transit union and current or former smokers. All research protocols were approved by the Institutional Review Boards of the Pacific Institute for Research and Evaluation and the University of California, San Francisco.

Fliers advertising the focus groups were posted around the bus garages’ and maintenance facilities’ Gillie (break) rooms. Project staff conducted on-site recruitment of participants with the following inclusion criteria: (1) current smokers (smoked some days or every day), (2) lapsed quitters (those who tried to quit but began smoking again in the last 6 months), or (3) those who had successfully quit while employed at the transit agency. Smoking status was ascertained by project staff administering a brief screening form to potential study participants.

Eleven focus group discussions were held over a 4-month period, with total of 71 workers (45% female; 83% African American) participating. Most participants (83%) were bus operators; the remainder consisted of maintenance workers or clerks (see Table 1). Approximately 67% of the participants were current smokers, and about 33% were former smokers. In terms of packs per day (PPD) smoked, the majority of current smokers (72%) reported less than 1 PPD; 21% reported 1 PPD; and 6% reported smoking at least 1.5 PPD. Among former smokers, about half (48%) reported smoking less than 1 PPD prior to quitting; 39% reported 1 PPD; and 13% reported at least 1.5 PPD. Current and former smokers participated jointly in focus groups. Workers were offered a $40 cash incentive fee for participating in a 90-minute moderated focus group discussion. Written informed consent was obtained from all participants.

**Table 1:** Socio-demographics and tobacco use among study sample*

**Demographics**	**N = 71**
	**N (%)**
Gender	
• Female	32 (45)
• Male	39 (55)
Age, years (SD)	49.9 (9.06)
Ethnicity	
• African American/Black	59 (83)
• Asian / Pacific Islander	3 (4)
• Hispanic/Latino(a)	5 (7)
• White	4 (6)
Job classification	
• Bus operator	59 (83)
• Mechanic / Maintenance	6 (9)
• Clerk / Dispatcher/ Other	6 (9)
Current Smoker (PPD) N = 47 (67%)	
• <1	34 (72)
• 1	10 (21)
• ≥1.5	3 (6)
• Smoking duration, Years (SD)	22.9 (12.30)
• Number of quit attempts (SD)	4.7 (7.85)
Former Smoker (PPD) N = 23 (33%)	
• <1	11 (48)
• 1	9 (39)
• ≥1.5	3 (13)
• Smoking duration, Years (SD)	18.7 (9.56)
• Number of quit attempts (SD)	3.6 (3.9)

*Smoking behaviors are missing for one focus group participant.

A semi-structured focus group interview guide was used to explore views and perceptions of smoking in general and in relation to work, knowledge of HMO smoking cessation treatment, and barriers to participation in cessation activities. Questions about perception to cessation included, “What do you know about smoking cessation activities and programs?” along with questions about barriers to participation in cessation activities such as, “How might job strain interfere with tobacco cessation activities?” The first of the 11 focus groups served as the pilot for testing questions and focus group procedures. During the pilot, observational notes were collected on participants’ responses, in addition to the administration of a brief participant survey to assess the validity of the questions. Finally, the team met immediately after the first group to review focus group questions to discern whether questions required adjustments.

Digitally recorded discussions were transcribed verbatim, and later focus groups’ transcripts were uploaded into the *NVivo*9 software program [14] to facilitate data management and analysis. After review of transcripts by the first three authors, the research team developed a coding manual, where data were first iteratively reviewed and discussed by members of the team to identify key themes, recurrent patterns, and specific perceptions of beliefs about cessation that were identified as most and least helpful. Later, the relevant sections of the transcripts were labeled with codes consisting of words or short phrases to summarize the central points of each paragraph [15]. All transcripts, including the pilot focus group, were coded by the researchers using a thematic analysis approach with the *NVivo* software program. Comparing coded segments of text across all transcripts helped the team to identify recurring patterns. Findings were discussed in research team meetings, with results being cross-validated between two team members to ensure reliability.

## Results

Content analysis revealed three major themes regarding beliefs and perceptions about smoking cessation. Table 2 highlights the major themes and their associated codes.

**Table 2:** **Extracted beliefs and perceptions regarding cessation**

**Extracted themes**				
**Readiness to quit**	**Addiction**		**Cessation experiences**	
***Associated codes***	***Associated codes***		***Associated codes***	
• Motivation	• Physiology & dependency	• Physiological make up	• Personal experience influences	• Observed experience influences
• Strength to quit				
	• Triggers	• Tobacco industry & chemicals	• Research on cessation type successes	

Readiness to quit. The most frequently discussed belief related to people being “ready to quit” when they had the motivation and strength to stop smoking. Some believed that even with cessation classes, only readiness to quit would lead to success.

*“I don’t really see that just having a class at a certain time is beneficial… It would be good to have it. But ultimately, nobody’s going to quit until they are ready to quit, make an effort to stop.”*

Addiction. Some of the respondents discussed their beliefs around smoking cessation as the habit being an addiction, remarking how one’s physiology played an important role with addiction.

*“It’s a chemical dependency. I mean it’s in your bloodstream. When everything is in the blood, you know that it will affect your whole body. Therefore, you know, the brain is already addicted to it, and the thinking just can’t go away.”*

Similarly, discussions around addiction included comments on the differences in the chemical composition of individuals’ bodies and the different responses one experiences when exposed to the chemicals found in cigarettes or alcohol. Some participants believed it was people’s physiological differences that made some more prone to addiction than others, or allowed some to have an easier time quitting than others.

Discussion of addiction at times focused on what participants deemed to be “triggers” for themselves or others, or common times or situations that triggered one to want to smoke.

*“… you can get into an altercation with somebody, or I got all this time on my hands, and it’s like, okay… So the brain triggers, and tells me ‘Oh, it’s time to have a cigarette.’ So, I go reaching to get a cigarette. Or, I can think of situations, and it’s like, ‘Oh, it’s time to get a cigarette.’”*

The term “trigger” was often used in association with work stress, life stress, or habitual practices, such as end of shift or break, free time, or after a meal. Triggers were also discussed in terms of the body being used to smoking, such as needing a cigarette upon awakening or under duress.

Others also mentioned the tobacco industry’s placement of chemicals into tobacco products to induce addiction. Several of the participants who commented on the tobacco industry’s role in addiction believed that such chemicals made it difficult to stop smoking.

*“If the cigarette manufacturers are putting stuff in the cigarettes that make your body addicted to ‘em, then how are you going to quit? I mean, your body needs the stuff because they put the stuff in cigarettes. So it’s going to be really hard to quit.”*

*“The companies that make tobacco, grow it, they know what to put, as scientists and all that. They have all these people. They know what influences the craving. Same with food. It’s a chemical that triggers another chemical in your body that keeps you craving for it. It’s worse than heroin.”*

Cessation experiences. Personal as well as observed cessation experiences appeared to play an important role in participants’ beliefs about quitting. Particularly, observed experiences seemed to influence whether participants believed any cessation program would help them stop smoking.

*“I’ve listened to people say they had hypnosis, but they still standing on the balcony [at the transit agency’s headquarters] smoking with me. So that didn’t work, so I don’t want to try that.”*

Beliefs about cessation were also influenced by direct or observed experiences with NRT and pharmacotherapy side effects. As one participant noted:

*“I tried the patch. I burned myself. I smoked anyways. I got the pills. Haven’t touched them because it’s going to give me nightmares.”*

Another participant noted his dislike of Wellbutrin, stating it was for *“people that’s kind of cuckoo.”* Those who experienced or observed negative effects of cessation therapies were less likely to believe it would help them quit.

Finally, some participants believed smokers might be more inclined to try different cessation methods if more research was made available about various cessation therapies’ success rates, or if they could observe successful cessation classes in progress:

*“… there would be more people interested if they see “Well, I went to this class and I haven’t smoked in two or three days,” then he might want to come and see what’s going on to stop them from smoking.”*

## Discussion and conclusion

Study findings suggest that among transit workers, smokers and former smokers recognize the need for people not to smoke, and are aware of various cessation programs and NRTs. These individuals also believed factors such as a smoker’s readiness to quit, recognition of the elements of addiction, and personal or observed experience with cessation methods facilitated successful cessation.

In a review of the Health Belief Model as a means for smoking cessation [9], such elements or personal beliefs about cessation were found to influence attitudes, which in turn influenced self-efficacy (e.g., belief of one’s own ability to quit smoking) towards behavior change [9,16]. A strongly related sentiment expressed by several participants was that one must believe they are ready to quit. Similarly, results related to beliefs based on the observation of others’ experiences with cessation related to the Social Cognitive Theory [10], which suggest observed modeled behavior and the consequences of that behavior are remembered and later used to guide subsequent behaviors. Such findings suggest that individuals do use others’ cessation experiences to determine how they feel or whether they believe in cessation. If participants do not believe the program will work based on modeled behavior, then they are less likely to try the cessation approach for themselves. Future cessation programs should take this into account, as this belief can actually form a barrier to participating in a cessation program.

Not only did current smokers believe that “I can quit when I am ready;” former smokers did as well, particularly those who quit “cold turkey.” These results fall within the realm of the Transtheoretical Model [11], which proposes that individuals’ readiness to start a new behavior or quit an old behavior through stages of change. Study results indicate that several of the current smokers were at the third or preparation stage, while former smokers had moved on to the fourth or “action” stage and were practicing the final or maintenance stage. As with application of the Social Cognitive Theory, future cessation programs should take into account participants’ beliefs about quitting and how these beliefs relate to the TTM’s stages of change. It is recommended that these programs also consider an emphasis on the model’s contemplation or pre-action plan stage, which takes into account stress management, a component that could possibly address the triggers discussed by participants as well maintenance sustained by the former smokers.

Additionally, beliefs about being ready to quit or quitting cold turkey may indicate the need for workers to gain an informed understanding about the ease of overcoming addiction, particularly to tobacco. Participants seemed to understand addiction in terms of physiology, recognizing the association between triggers and the body’s craving for nicotine. Some of the participants were curious about cessation, yet their beliefs about addiction, and observations of others’ failures to quit, led many to believe cessation was hopeless. Developers of cessation programs must include accurate information about addiction, which in turn may alter people’s beliefs about smoking cessation. Documenting success rates of different cessation programs may provide a reality check for such beliefs, thus encouraging quitting intentions.

Given participants’ often cynical beliefs about cessation, it would at first glance appear that existing cessation programs do not fully meet their needs. Perusal of the HMO’s cessation site found an online program where after one answers a series of questions, a personalized program is provided to the participant. Of interest is that within the personalized program, as well as on the cessation site, there are strategies for addressing what participants noted as readiness to quit, where tips were provided for preparing to quit. Additionally, strategies for coping with stress were available. Such strategies could help with participants concerned with triggers. Finally, there were recommendations for dealing with withdrawal, something that could help address participants’ concerns about addiction. Review of the HMO cessation site indicates there are strategies that directly address participants’ beliefs about cessation. Such information is encouraging; however, a challenge study participants face, in addition to their beliefs, is the challenge of the workers’ temporal availability to take part in cessation activities.

Many of the study’s participants noted they needed more flexible time to participate in any type of cessation program [6]. While a personalized online program seems less time intensive than a weekly class, it is still necessary for the HMO to consider how it can bring its cessation programming “closer” to the participants. Given participants beliefs about cessation coupled with time challenges, it would benefit the HMO and its cessation program developers to devise a strategy for making the online services more readily available, such as having kiosks available at the worksites. Alternatively, they might consider hosting monthly sessions at the worksite where the content simply includes how to use one’s smartphone to access the online cessation program. The goal is to connect participants with a cessation program that addresses their beliefs, thus additional approaches must be further investigated.

Concerns about tobacco additives and the side effects of NRTs among largely African American participants were similar to findings from a study on beliefs about smoking cessation among young African Americans [17]. Young adults from this study expressed disbelief about efficacious smoking cessation programs. Such statements may imply a lack of information about cessation programs. These concerns may also reflect mistrust of the medical establishment, common within the African American community [18-21]. Implications of these findings indicate that notions of mistrust may lead to misinformation, which in turn can hinder participation in cessation programs. Cessation program personnel should strive to be mindful of these factors when promoting their programs to potential participants as well as providing them with an informed understanding about smoking cessation methods, including NRT and pharmacotherapy. Additionally, future studies investigating cessation among high-risk populations, such as transit workers who include a large percentage of individuals heavily impacted by tobacco-related health inequities [8], should explore this phenomenon of mistrust. Given the history of mistrust among many African Americans as a result of either personal discrimination or knowledge of unethical practices [20-22], further exploration of how to address these perceptions may aid in developing strategies for increasing cessation among African Americans. Furthermore, understanding how such perceptions affect individuals’ attempts at cessation can enhance cessation efforts and improve chances of success.

Strengths and Limitations. The current study adds to the literature by addressing barriers to cessation among a sample of urban transit workers, an occupational group with elevated smoking prevalence [2,4,23]. An important strength of the study is that it qualitatively analyzed how transit workers’ beliefs may be associated with choice to quit smoking or the selection of a cessation method. Understanding such beliefs lays the foundation for future studies with this population as it relates to implementing cessation programs or working with local unions and HMOs making concerted efforts to increase participation in the HMO’s cessation programming.

As mentioned earlier, one of the major stressors faced by transit workers is schedule-related time pressures. This includes non-standard work schedules, split shifts, being on call, or having to maintain a tight fixed schedule while out in the field. Such challenges made it difficult to host smoker-specific focus groups; thus, current and former smokers were combined in the same focus group sessions to accommodate job-related time restrictions. While there was concern about former smokers hesitancy to speak amongst smokers, this was not the case; we found that being in the same group enabled important dialogue and exchange among workers who have grappled with tobacco cessation at different stages of change to take place.

It is important to consider the findings within the context of existing research on transit workers. Most of the studies have focused on transit operators (typically bus operators, but also light rail or streetcar operators), and show significant associations between occupational stressors and numerous physical, psychological and behavioral health outcomes [3,24]. Elevated rates of current smoking relative to the general population have been reported among transit workers in San Francisco [25] and Minneapolis [23]. In the former study, frequency of job problems (e.g., difficulties with equipment, passengers, traffic) was related to likelihood of smoking increase, initiation, or maintenance over a 10-year period [4]. Workers who reported smoking as a coping strategy were more likely to report job burnout symptoms, drink alcohol, and smoke [5]. In addition to our recent qualitative study [6], the current study is among the first to explore objective and perceived barriers to cessation among transit workers. Given the tobacco-related disparities among this occupational group, the findings herein help fill a critical gap in knowledge that can be used to increase transit worker participation in cessation activities that are available to them as an employee benefit.
